# Anti-breast Cancer Activity of SPG-56 from Sweet Potato in MCF-7 Bearing Mice *in Situ* through Promoting Apoptosis and Inhibiting Metastasis

**DOI:** 10.1038/s41598-018-29099-x

**Published:** 2019-01-16

**Authors:** Zhaoxing Li, Yang Yu, Meimei Wang, Heshan Xu, Bing Han, Pu Jiang, Hang Ma, Yuanfeng Li, Cheng Tian, Deqi Zhou, Xuegang Li, Xiaoli Ye

**Affiliations:** 1 School of Pharmaceutical Sciences, Southwest University, Chongqing, 400716 China; 2 McLean Hospital, Harvard Medical School, Belmont, 02478 MA USA; 3 Chongqing Engineering Research Centre for Sweet Potato, School of Life Sciences, Southwest University, Chongqing, 400715 China; 4 Oncology Department, Chongqing Beibei District Hospital of Traditional Chinese Medicine, Chongqing, 400700 China

## Abstract

SPG-56 is a newly isolated glycoprotein from sweet potatoes (Zhongshu NO. 1), but its value for suppressing breast cancer progression remains unknown. This study was designed to investigate the potential anti-cancer effects of SPG-56, which consists of 2.9% sugar and 97.1% protein. The effects of SPG-56 on the proliferation and apoptosis of breast cancer cells were determined using CCK-8 and Hoechst 33342 assays and flow cytometry, after staining with Annexin V and PI respectively. The activities of SPG-56 against breast cancer were examined using female BALB/c nude mice orthotopically implanted with human breast carcinoma cells of the types MCF-7 and 4T1-Luc. The cellular experiments showed that SPG-56 inhibited proliferation and promoted apoptosis of MCF-7 cells dose- and time-dependently. Oral administration of SPG-56 significantly suppressed the development of MCF-7 tumor cells (P < 0.01) as compared with an untreated group. The serum tumor markers CEA, CA125 and CA153 in a 240 mg/kg/d SPG-56 decreased by 54.8%, 91.8%, and 90.3%, respectively. The experiments further demonstrated that SPG-56 inhibited the metastasis of breast cancer in MCF-7 and 4T1-bearing mice by altering the expression of MMP2, MMP9, VEGF, Occludin and Claudin. It is concluded that SPG-56 may have potential as a novel anti-tumor candidate for breast cancer.

## Introduction

Breast cancer is the second leading cause of cancer-related deaths around the world^1^, as well as the most common type of cancer in China^2,3^.

Induction of apoptosis is one of the most important mechanisms by which an anti-tumor agent works^4^. Apoptosis can be induced via two pathways, an extrinsic death-signal-induced receptor-mediated pathway, or an intrinsic distress-induced mitochondrion-mediated pathway^5,6^.

Modern research results show that the sweet potato (*Ipomoea batatas* (L.) Lam.) and its active ingredients have the effect of enhancing immunity^7^, anti-oxidation^8^, inhibiting inflammation and inhibiting cardiovascular diseases^9,10^. Anti-cancer ingredients in sweet potatoes mainly include polysaccharides, sporamin, DHEA, anthocyanin and glycoprotein^11–13^. As a type of mucoprotein, sweet potato glycoprotein is an important biological active constituent of sweet potatoes, with health-promoting functions and medicinal properties. Glycoprotein have pharmacologic actions such as anti-cancer^14^, anti-oxidant^15^, anti-hyperlipidemia^16^, hypoglycaemic^17^ and immunological enhancement^18^. Recently, Wang *et al*. extracted and isolated sweet potato glycoprotein-56 (SPG-56) at molecular weight of 56000, consists of 2.9% sugar and 97.1% protein, which was found to enhance the apoptosis of colon cancer cells^19^.

In this study, nude mice were used as experimental animals to evaluate the anti-breast cancer effects of SPG-56 *in vivo* and *in vitro*. To clarify its possible molecular mechanisms, the effects on serum inflammation, tumor markers, and related proteins were investigated. The results are expected to provide a theoretical and experimental basis for the use of SPG-56 in anti-breast cancer applications in the future.

## Materials and Methods

### Materials

SPG-56, supplied by our laboratory in Southwest University, was isolated from Zhongshu-1 according to previous described methods^19^. The detail of SPG-56 was shown in the supplement file.

### Cell Culture

Human breast cancer cell line MCF-7 and mouse breast cancer cell line 4T1-Luc (Shanghai, China), which ransfected with firefly luciferase, were incubated in Dulbecco’s Modified Eagle Medium (DMEM), supplemented with 10% fetal bovine serum (GIBCO, New Zealand) and 1% penicillin/streptomycin, while human breast continuous epithelial cell line HBL-100 (Cell Bank of Shanghai Institute for Biological Sciences, Shanghai, China) was cultured in 1640 Medium (1640) supplemented with 10% fetal bovine serum and 1% penicillin/streptomycin. All cells were maintained in a humidified atmosphere with 5% CO_2_, at 37 °C.

### Cell Proliferation Assay

HBL-100 cells were used as a control to detect the toxicity of SPG-56 to normal cells. MCF-7 and HBL-100 cells were transferred into 96-well culture plates at a density of 6000 cells per well. After incubation overnight, the cells were treated with specified concentrations of SPG-56 (5 to 320 μg/ml for MCF-7 and HBL-100 cells) for 24 h at 37 °C. The effect of SPG-56 on cell proliferation was analyzed using Cell Counting Kit-8(Genview, USA), according to the standard procedure.

### Cell Apoptosis Assay

MCF-7 cells or HBL-100 cells were seeded in triplicate into 12-well plates at a density of 10^5^ cells/well and grown for 24 h. The cells were then treated with 104.14 μg/ml of SPG-56 or PBS for 12, 24, 36 and 48 h, respectively. The number of viable cells was determined using the Trypan blue exclusion assay(Dinguobio, Beijing, China), according to the manufacturer’s instructions.

### Apoptosis Activity Analysis of SPG-56

MCF-7 cells (4 × 10^5^ cells/well) were treated with various concentrations of SPG-56 (0, 10, 20 μg/ml) for 24 h. Cell apoptosis was detected using an Annexin V and propidium iodide (PI) kit (Wanleibio, Shenyang, China) according to the manufacturer’s instructions. The stained cells were subjected to a BD FacsVantage SE Flow Cytometer (BD Biosciences, San Jose, CA, USA). The data were analyzed using Flow Jo 7.6.1 software (Tree Star Inc., Ashland OR, USA).

### Hoechst 33342 Staining

After treatment in 12-well plates for 24 h, cells were stained with the dye Hoechst 33342(Dinguobio, Beijing, China) at 37 °C for 15 min, in the absence of light. The cells were then washed with PBS 5 times, and images were captured using a Nikon fluorescence microscope (Nikon, Japan).

### Preparation of *in situ* Inoculation Model of Tumor

Healthy four-week-old female BALB/c nude mice with a weight of 14 ± 1 grams were permitted to feed and drink freely. The animal experiments were formally approved by the Institutional Animal Care and Use Committee of Southwest University.

After one week of *ad lib* feeding, the fifty nude mice were randomly divided into five groups, comprising normal controls (NC), tumor controls (TC), SPG-56 with a low dose of 60 mg/kg body weight (SPG-L), SPG-56 with a middle dose of 120 mg/kg body weight (SPG-M), and SPG-56 with a high dose of 240 mg/kg body weight (SPG-H).

Prepared MCF-7 cell suspensions (30 μl matrigel and 90 μl of PBS containing 10^6^ cells) were orthotopically implanted into the nude mouse breast pad^20^. From the first day of inoculation, mice in the NC and TC groups received appropriate amounts of 0.9% saline solution. SPG-56 at doses of 60, 120 and 240 mg/kg of body weight was administered daily by gavage. Tumor growth was traced every two days by vernier caliper along two orthogonal axes, length (L) and width (W). The volume (V) of a tumor was calculated using the equation for an ellipsoid (V = W^2^*L/2). The body weight of each mouse was measured every two days. On the final day, blood samples were collected for a multiple tumor marker protein biochip assay, and the tumors were quickly dissected away from surrounding tissue and weighed, then subsequently stored at −80 °C.

### Analysis of Serum Tumor Markers and Inflammatory Factor

A Multi-tumor marker protein chip diagnosis system was used to detect the tumor markers CEA, CA125, and CA153 at Chongqing Cancer Hospital (Chongqing, China). ELISA kits were used to measure IL-6 (Multi Sciences, Hangzhou, China), TNF-α (Multi Sciences, Hangzhou, China), and NFκB (Cloud-Clone Corp., TX, USA) levels in the serum.

### HE Staining in Liver

The fresh livers of the mice were cut into pieces, each side of which measured 0.3 cm–0.5 cm. These cubical pieces were fixed with 10% paraformaldehyde for 24 hours, then dehydrated using a graded ethanol series. Xylene I and II were applied for 20 min for clearing, followed by wax immersion and embedding of the pieces. The pieces were then sliced (4 μm thick), dewaxed, and Hematoxylin and Eosin (HE) stained, for examination of pathological changes.

### Changes in Protein Expression by Western Blotting

The expression of Caspase-3, Caspase-8, Bcl-2, Bax, MMP2, MMP9, VEGF, Occludin, Claudin and STAT3 from SPG-56 treated mice tumors, and the expression of MMP2, MMP9, VEGF, Occludin, Claudin and STAT3 from control livers, were analyzed by western blotting. The procedure for immunoblotting followed a previous report^21^. Total protein was extracted from the cells by 1X RIPA buffer (BBI life sciences, China) containing 1 mM phosphatase inhibitor cocktail and 1 mM phenylmethylsulfonyl fluoride (PMSF). Equalamounts of proteins (10 μg) were subjected to SDS-PAGE. After transferred onto a polyvinylidene fluoride membrane, they were incubated with primary antibodies against β-actin, Caspase-3, Caspase-8, Bcl-2, Bax, VEGF, Occludin, Claudin, STAT3(Proteintech Group, Inc., USA), MMP2 and MMP9(Absin Bioscience Inc., China), respectively (1:1000 dilution, 4 °C, overnight). Western blot was quantified by using ImageJ software.

### Immunohistochemistry of Caspase-3, Caspase 8, Bcl-2, Bax and MMP9

Changes in Caspase-3, Caspase 8, Bcl-2, Bax and MMP9 expression in the untreated control and drug treated tumor tissues, and MMP9 expression in untreated control and drug treated livers, were evaluated by immunohistochemistry (IHC) using specific antibodies directed against each target protein. The procedure for immunohistochemistry followed a previous report^19^. Tissue sections (8 μm) were cut. Endogenous peroxidase activity was quenched by adding 3% H_2_O_2_ for 15 min. Then, sections were placed in 10% normal goat serum for 1 h to block non-specific binding. Apoptosis was evaluated using a polyclonal anti-cleaved Caspase-3 antibody (rabbit polyclonal, 1:500 dilution), further incubated with a HRP-conjugated secondary antibody for 1 h at room temperature. And the same procedures to Caspase-8, Bcl-2 and Bax. After PBS washing, chromogenic signal development was developed using 3, 3′-diaminobenzidine tetrahydrochloride in 50 mmol Tris-HCl (pH 7.5) for 5 min. Counter staining was carried out using hematoxylin and the sections were visualized under light microscope.

### Luminescence Imaging for Detection of Metastasis *in Vivo*

Using the same animal breeds and feeding methods described above, twelve 4T1-Luc breast cancer bearing mice were randomly divided into two groups, 4T1 tumor controls (4T1-TC) and SPG-56 subjects given a high dose of 240 mg/kg of body weight (4T1-SPG). Twenty days after inoculation, the mice were injected with 10 μl/g D-Luciferin (Yesen, Shanghai, China), Potassium Salt via the tail vein. Ten minutes later, the mice were anesthetized by intraperitoneal injection of 10% chloral hydrate, and the distribution of luminescence in the mice was scanned using a VILBER FUSION FX7 Spectra multifunctional imager (VILBER LOURMAT, France), at a wavelength of 560 nm.

### Statistical Analysis

The experimental data were processed using the statistical analysis software SPSS 20.0. Variables were expressed as mean ± standard deviation (SD), and statistical analysis was performed using a one-way analysis of variance (ANOVA). P < 0.05 and P < 0.01 were distinguished as statistically significant.

### Ethical standards

All procedures performed in this study were in accordance with the ethical standards of the national independent ethics committees and institutional review boards of the study centers. The study was conducted in accordance with Good Clinical Practice and all applicable regulatory requirements, including the Declaration of Helsinki.

## Results

### SPG-56 Inhibited the Proliferation of MCF-7 Cells, but Only Minimally Influenced the Growth of HBL-100 Cells

**Figure 1:**
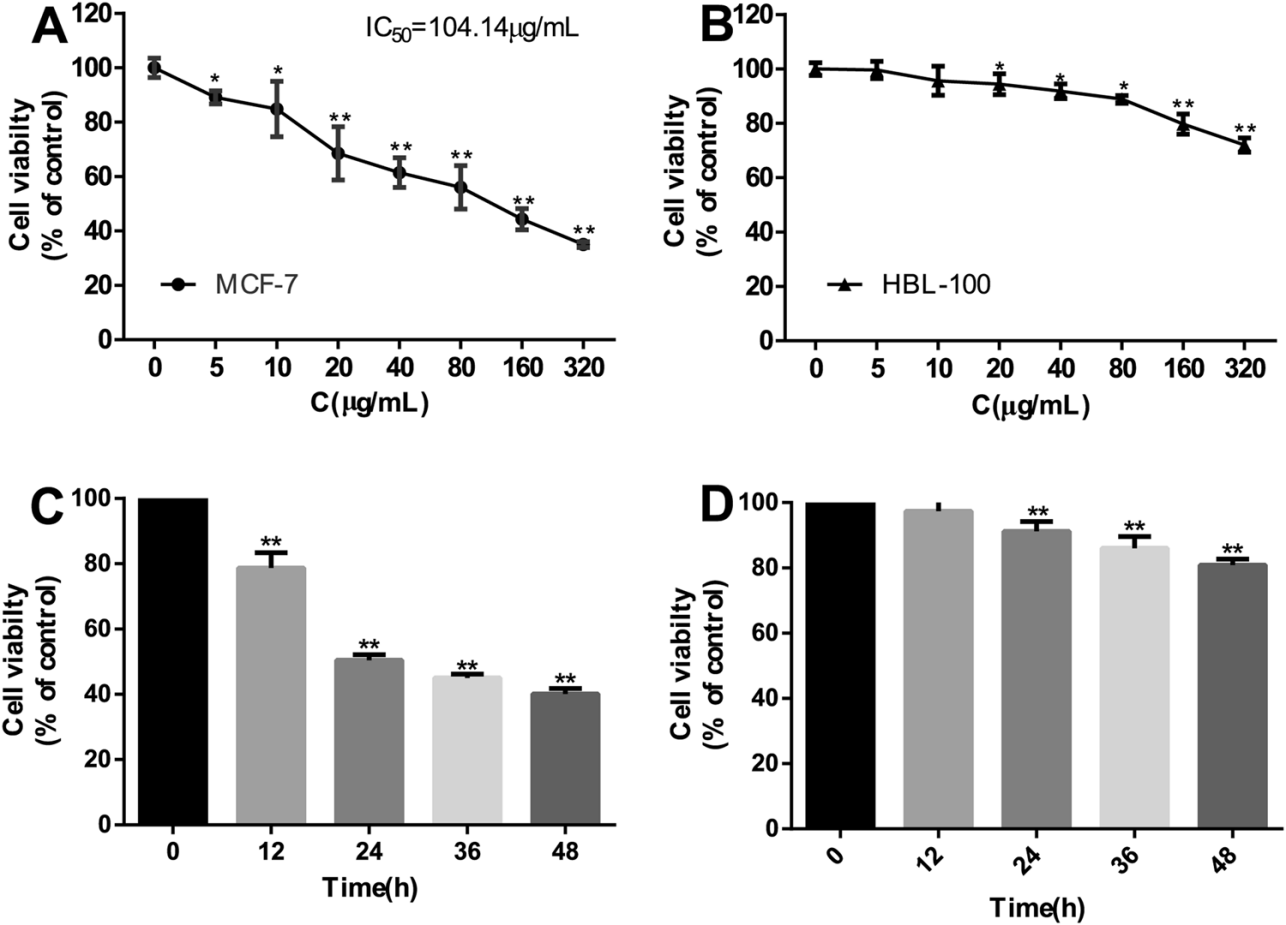
SPG-56 induced different cell death in MCF-7 cells and HBL-100 cells. (**A**) MCF-7 cells and (**B**) HBL-100 cells were treated with indicated concentrations of SPG-56 for 24 h and cell viability was determined by CCK-8 assay. (**C**) MCF-7 cells and (**D**) HBL-100 cells were treated with 104.14 μg/mL of SPG-56 for 0 to 48 h, and counted using a hemocytometer. Values represent mean ± SD with six replicates. *p < 0.05 and **p < 0.01 compared with control.

To test the effectiveness of SPG-56 on MCF-7 cells *in vitro*, cytotoxicity and proliferation were determined by CCK-8 assay and Trypan blue exclusion assay, respectively. In addition, human breast epithelial cells (HBL-100), considered as normal cells for this purpose, were used to determine the cytotoxic effect of SPG-56. As shown in Fig. 1A,C, SPG-56 inhibited MCF-7 cell proliferation in a time- and dose-dependent manner. The IC50 of SPG-56 on MCF-7 cells was 104.14 μg/ml. However, SPG-56 treatment induced much less cytotoxicity in HBL-100 cells. Based on these results, we chose an optimal concentration range (1–40 μg/ml) and treatment time (24 h) for further *in vitro* studies. For further experiments, we used a dose of the drug that resulted in a cell viability of about 80%. We did this because it is important to use viable cells to effectively study the cellular effects of drug addition.

### SPG-56 Induced Apoptosis of MCF-7 Cells

**Figure 2:**
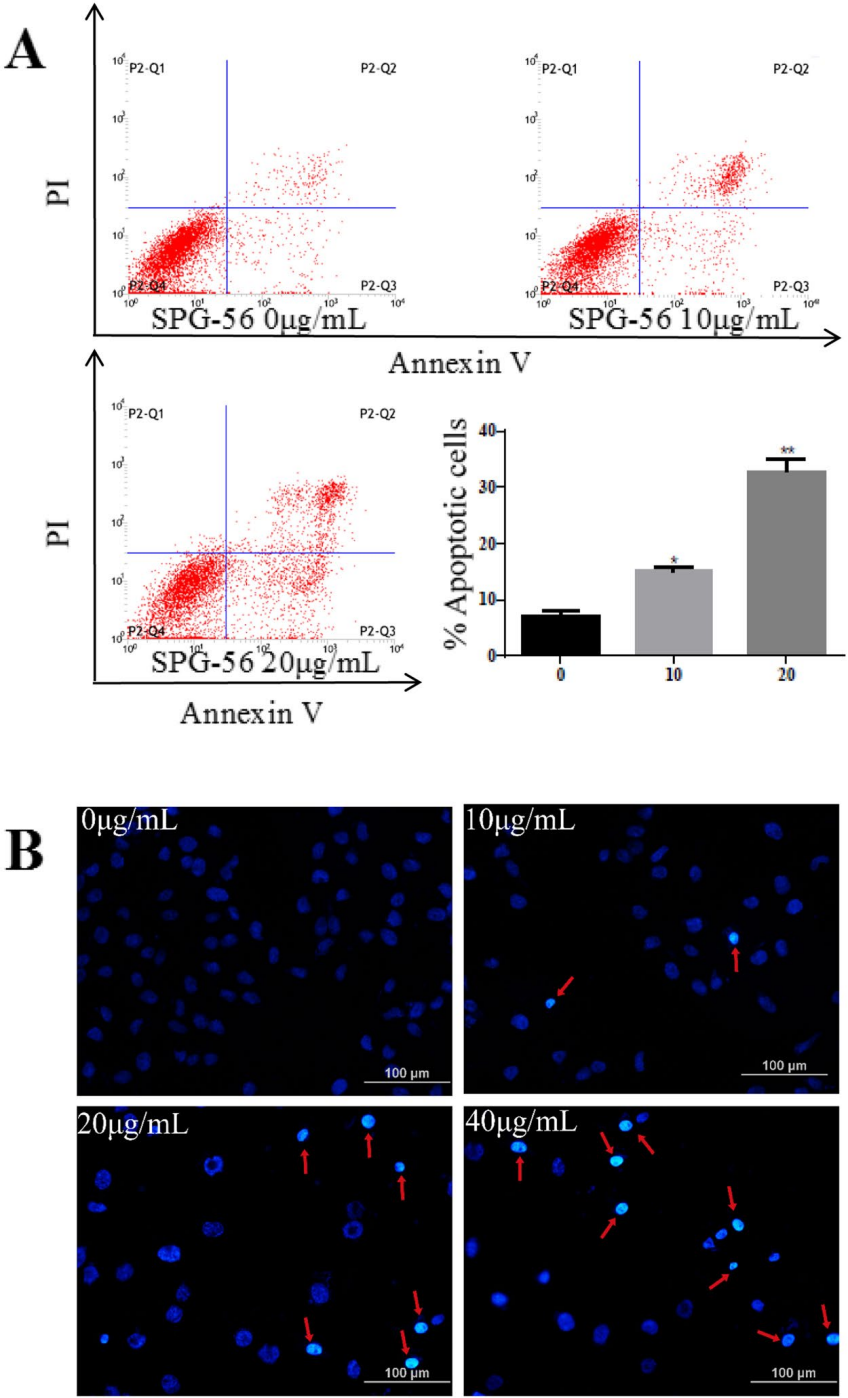
SPG-56induced apoptosis on MCF-7 cells. (**A**) Apoptosis cells were quantified by flow cytometry with Annexin V and PI. Representative scatter plots of PI (y-axis) versus annexin V (x-axis). Values represent mean ± SD with three replicates. *P < 0.05, **P < 0.01, compared with control. (**B**) Morphologic change of apoptotic cells was evaluated by Hoechesst 33342 staining. The scale bar was 100 μm.

The effect of SPG-56 on MCF-7 cell apoptosis was determined by staining cells with annexin V-EGFP/PI. Figure 2A shows that SPG-56 induced apoptosis in a dose-dependent manner. After treatment with 10 μg/ml SPG-56 for 24 h, the apoptotic rate of the cells increased to 15.0 ± 1.0%, as opposed with to 7.14 ± 1.1% of normal control cells. With an increase in treatment dosage to 20 μg/ml, the percentage of apoptotic cells was markedly increased, reaching 32.8 ± 2.5%. This suggests an anti-breast cancer pathway of SPG-56 mediated by induction of apoptosis.

Hoechst 33342 staining was used to examine morphological changes of the apoptotic cells. As shown in Fig. 2B, SPG-56 treatment at three dosages produced many more cells with highlighted, condensed, and fragmented nuclei than could be seen for the untreated controls.

### SPG-56 Inhibited Tumor Growth in MCF-7 Bearing Nude Mice *in situ*

**Figure 3:**
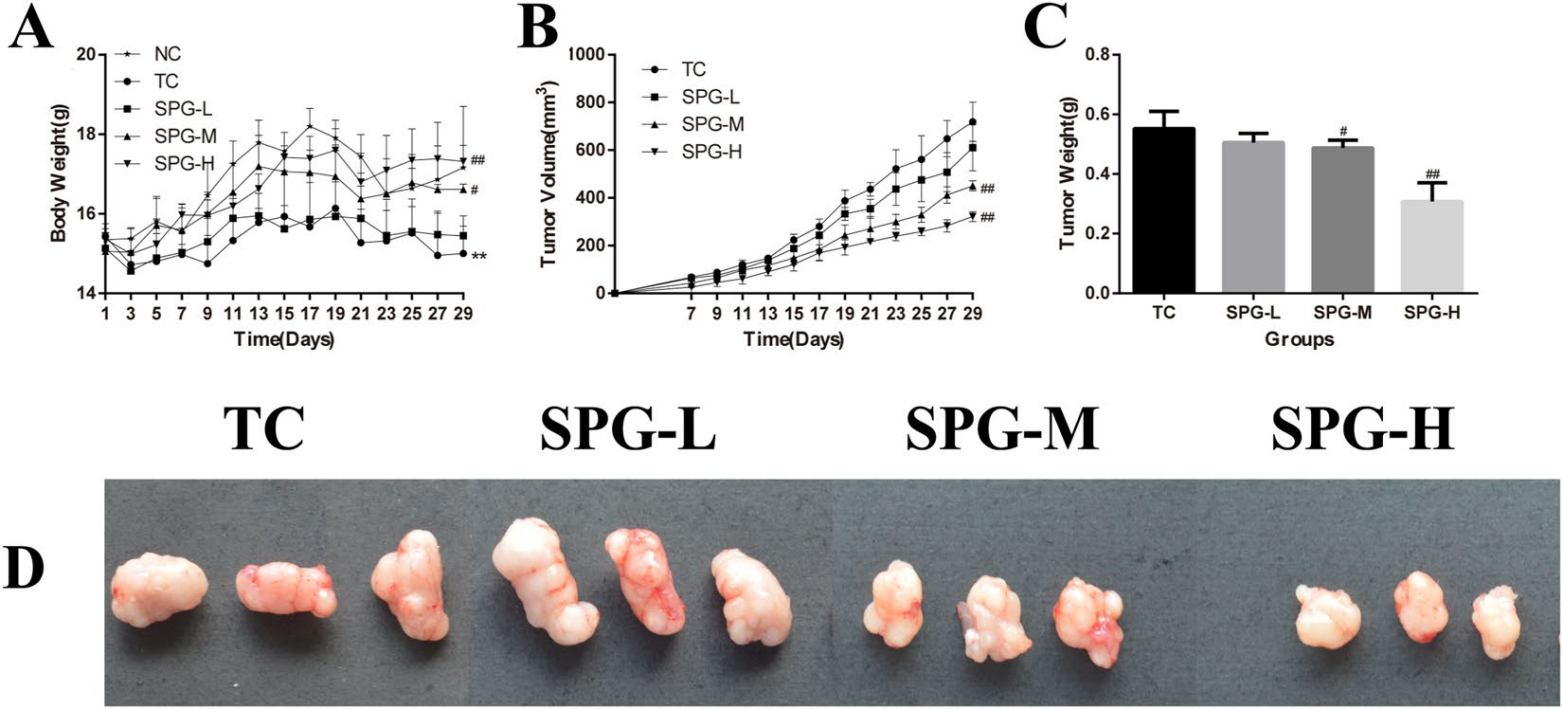
The effect of SPG-56 on the body weight and tumor growth of mice. (**A**) Effect on body weight; (**B**) Effect on tumor growth, manifested by tracking tumor volume; (**C**) Tumor Weight; (**D**) Tumor Tissues^*^P < 0.05, ^**^P < 0.01, compared with the NC group. ^#^*P* < 0.05, ^##^*P* < 0.01, n = 10, compared with the tumor control (TC) group.

After mice were injected with MCF-7 cells for 7 days, their body weights, and the volume and weight of tumors, were recorded every 2 days, as shown in Fig. 3. In the TC group, the volumes of the tumors gradually increased while the body weights decreased, as compared with the NC group. After treatment with SPG-56, the body weights of the mice increased dose-dependently in comparison to the TC group. The body weights of the SPG-H (high dose of SPG-56) group were 15.43% higher than the TC group (P < 0.01), while the body weights of the TC group were 17.03% lower than the NC group (P < 0.01). The mean tumor volume and mass for the TC group were 719.0 ± 79.0 mm^3^ and 0.552 ± 0.029 g respectively 29 days after injection with MCF-7 cells. Also, the spines of mice in the TC group appeared to be bent, and some of the tumors began to show rupture necrosis. SPG-56 inhibited the growth of the MCF-7 tumors in a dose-dependent manner. By the end of the experiment, the tumor volume and mass for the SPG-H Group were significantly smaller than for the TC group (322.0 ± 23.1 mm^3^, 0.307 ± 0.069 g, P < 0.01). This suggests that SPG-56 was able to suppress the development of MCF-7 tumor cells in mice.

### SPG-56 Reduced Tumor Markers in MCF-7 Bearing Nude Mice

**Figure 4:**
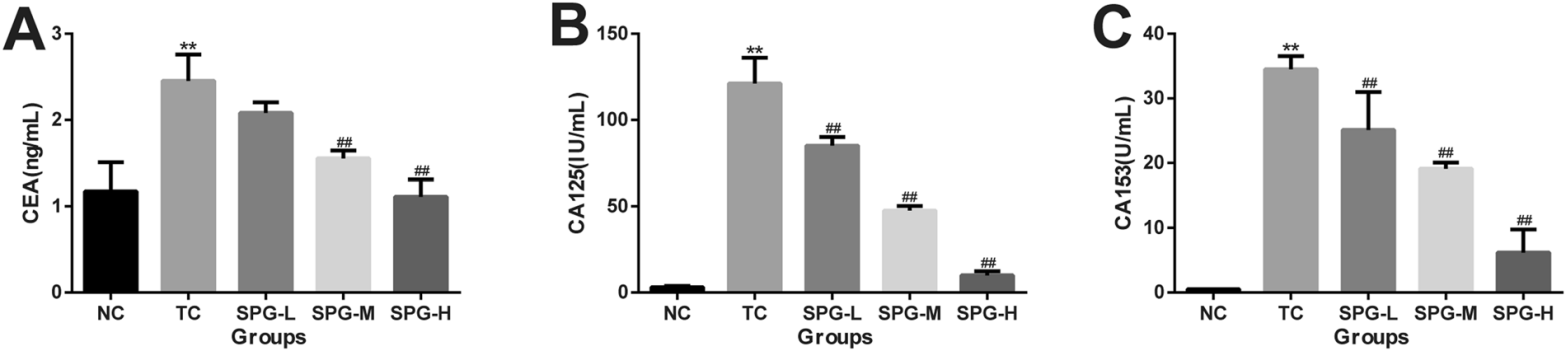
The effect of SPG-56 on the serum tumor markers. (**A**) CEA; (**B**) CA125; (**C**) CA153; ^*^P < 0.05, ^**^P < 0.01, compared with the NC group. ^#^*P* < 0.05, ^##^*P* < 0.01, n = 10, compared with the TC group.

Serum tumor markers, including CEA, CA153 and CA125, are commonly measured to diagnose the occurrence and prognosis of breast cancer. As shown in Fig. 4, the levels of CEA, CA153 and CA125 in the TC group were all significantly higher than for the NC group (P < 0.01). In comparison to the TC group, SPG-56 treatment reduced the levels of CEA, CA153 and CA125 significantly, especially in the high dose group (P < 0.01). These results indicate that SPG-56 slowed the development of tumor as tracked by multiple measurements.

**Figure 5:**
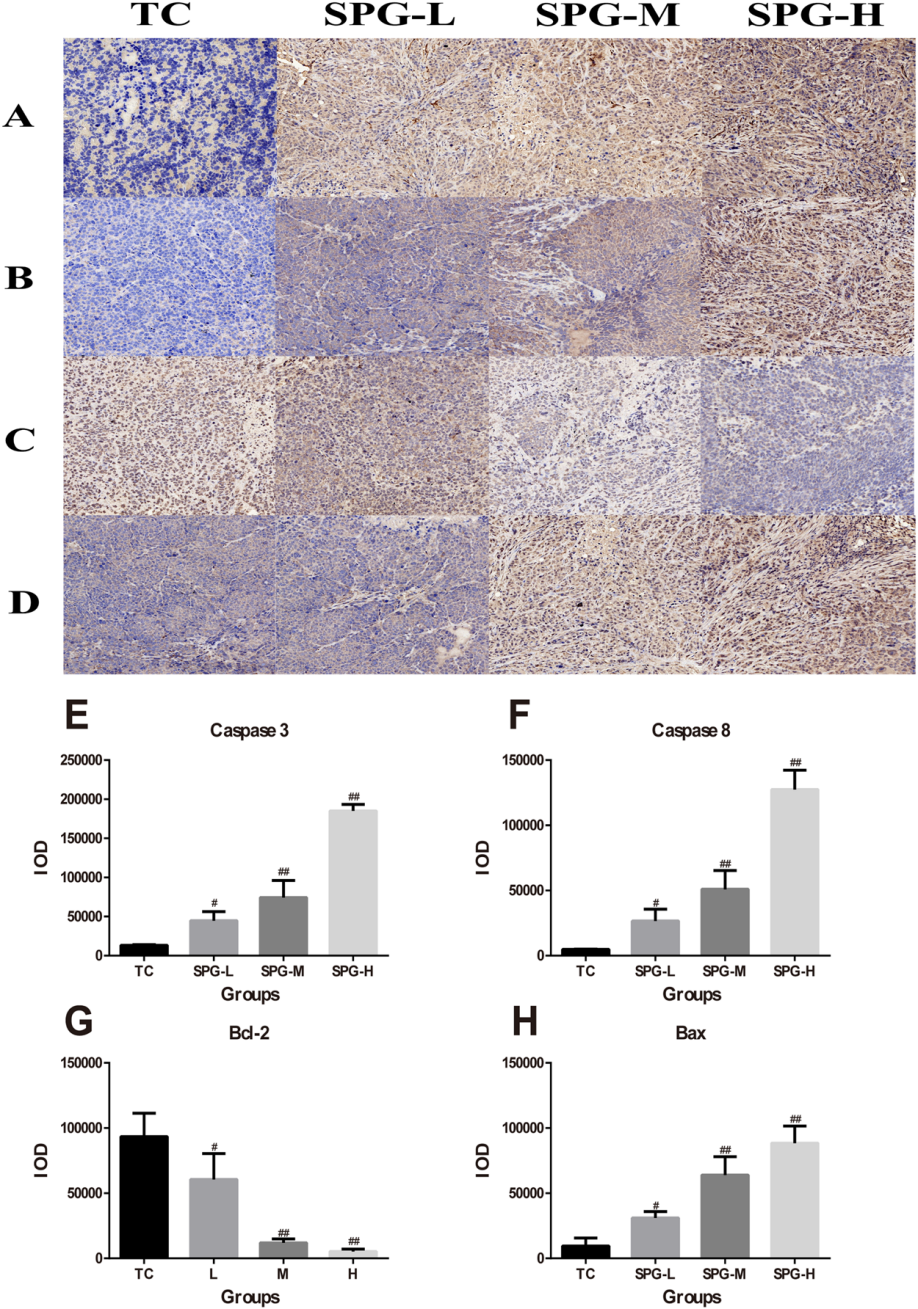
Effect of SPG-56 on apoptosis-related proteins in tumor. Image magnification: 20x. (**A**) Caspase-3; (**B**) Caspase-8; (**C**) Bcl-2; (**D**) Bax. IOD statistical analysis of immunohistochemistry of (**E**) Caspase-3; (**F**) Caspase-8; (**G**) Bcl-2; (**H**) Bax. Values represent mean ± SD with three Replicates. The data represent three similar experiments. **p < 0.01 compared with NC. ^##^p < 0.01 compared with TC.

**Figure 6:**
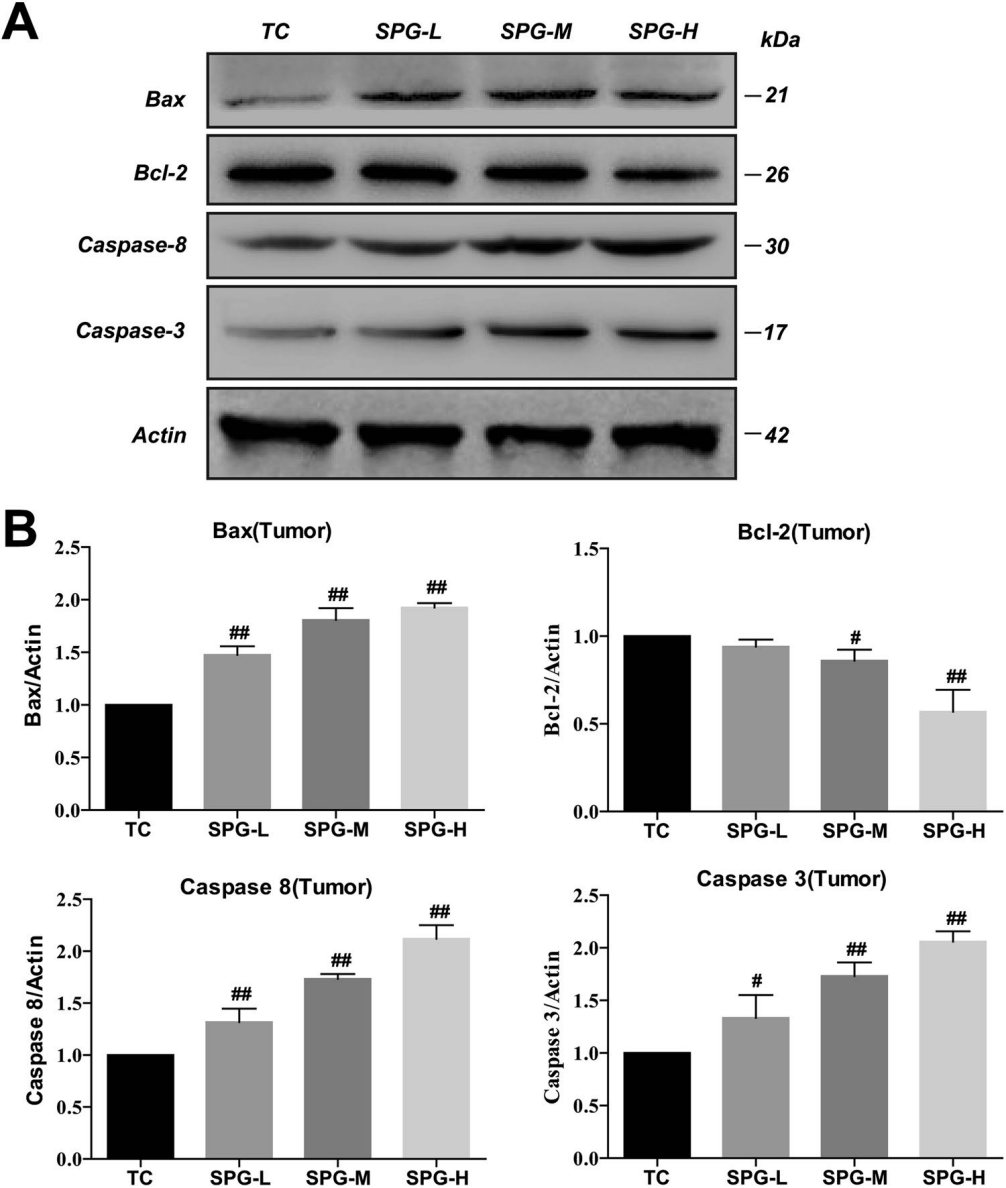
Effects of SPG on apoptosis-related proteins. The expression level of proteins were determined by Western Blotting which were a representative of three independent experiments. (**A**) The expression levels of Caspase-3, Caspase-8, Bcl-2, Bax and β-actin extracted from tumor tissues. (**B**) Statistical Analysis of Caspase-3, Caspase-8, Bcl-2, and Bax extracted from tumor tissues. ^#^*P* < 0.05, ^##^*P* < 0.01, n = 10, compared with the TC group.

Furthermore, western blotting and an immunohistochemistry assay showed that the levels of Caspase-3, Caspase-8 and Bax in the tumor tissues were significantly increased, while the level of Bcl-2 decreased in a dose-dependent manner (Figs 5 and 6). This suggests that SPG-56 could induce tumor apoptosis through a mitochondrial pathway, which accords with the effects of SPG-56 on MCF-7 cells (Fig. 2).

### SPG-56 Inhibited Cancer Metastasis in MCF-7 and 4T1-Luc Bearing Nude Mice

**Figure 7:**
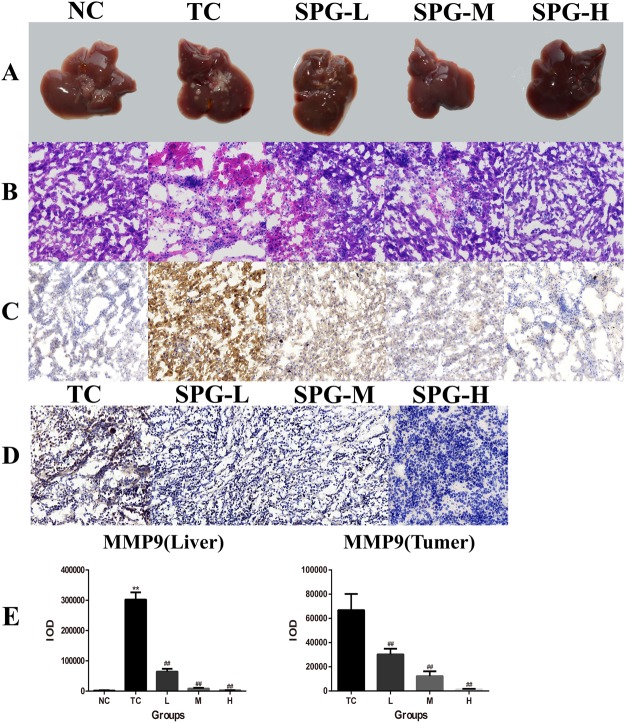
Effect of SPG-56 on the livers and tumors in MCF-7 bearing mice. (**A**) Representative pictures of livers in mice from these three groups harvested on the day 29 after the injection of cancer cells were shown. (**B**) Representative H&E staining sections of the livers from Fig. 5A. (**C**) Effect of SPG-56 on the MMP9 expression changes of livers. (**D**) Effect of SPG-56 on the MMP9 expression changes of tumors. The data represent three similar experiments. Image magnification: 20.0x. (**E**) IOD statistical analysis of immunohistochemistry of MMP9. Values represent mean ± SD with three replicates. **p < 0.01 compared with NC. ^##^p < 0.01 compared with TC.

At the end of experiment, we found that the livers of the TC group showed large-scale metastasis of cancer as shown in Fig. 7A. In contrast, the livers from mice treated with SPG-56 cells showed fewer lesions. Especially in the high-dose group, it was barely possible to observe metastasis of MCF-7 cells in the livers.

**Figure 8:**
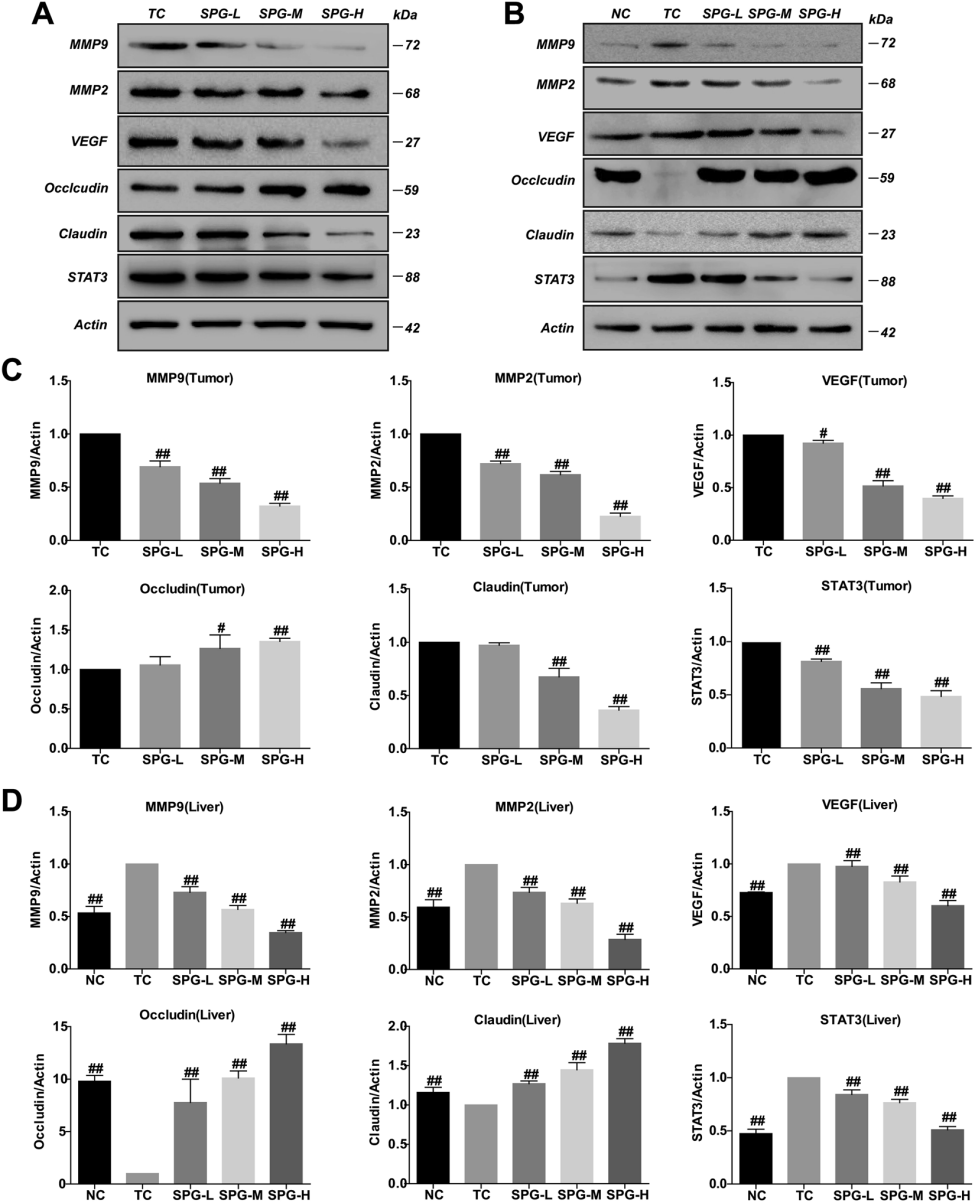
Effects of SPG on metastasis-related proteins. The expression level of proteins were determined by Western Blotting and the blots were a representative of three independent experiments. (**A**) The expression levels of MMP2, MMP9, VEGF, Occludin, Claudin, STAT3 and β-actin extracted from tumor tissues. (**B**) The expression levels of MMP2, MMP9, VEGF, Occludin, Claudin, STAT3 and β-actin extracted from liver tissues. (**C**) Statistical analysis of MMP2, MMP9, VEGFA, Occludin, Claudin and STAT3 extracted from tumor tissues. (**D**) Statistical analysis of MMP2, MMP9, VEGFA, Occludin, Claudin and STAT3 extracted from liver tissues. ^#^*P* < 0.05, ^##^*P* < 0.01, n = 10, compared with the TC group.

The results of HE staining demonstrated the occurrence of metastasis (Fig. 7B). Immunohistochemistry analysis showed that the expression of MMP9 in the TC group was significantly higher than in the NC group. However, administration of SPG-56 strongly reduced the expression of MMP9 in both the liver and the tumor dose-dependently, as shown in Fig. 7C–E. In the high dose groups, the expression of MMP9 in the tumors and livers were lowered by 97.9% and 99.1% compared to the TC group. The effective regulation by SPG-56 of the expression of metastasis-related proteins MMP2, MMP9, VEGF, Occludin, Claudin and STAT3 further confirmed the inhibition by SPG-56 of tumor metastasis (Fig. 8). Especially compared to the NC group, the expression of MMP2 in the livers of the TC group was significantly higher, and the expression of Occludin in the livers of the TC group was significantly lower. These findings could explain why the metastasies of the tumors in the liver were serious. The trend of protein expression in the drug group was just the opposite, especially in the middle and high dose groups, and some protein expression levels were indistinguishable from the NC group.

**Figure 9:**
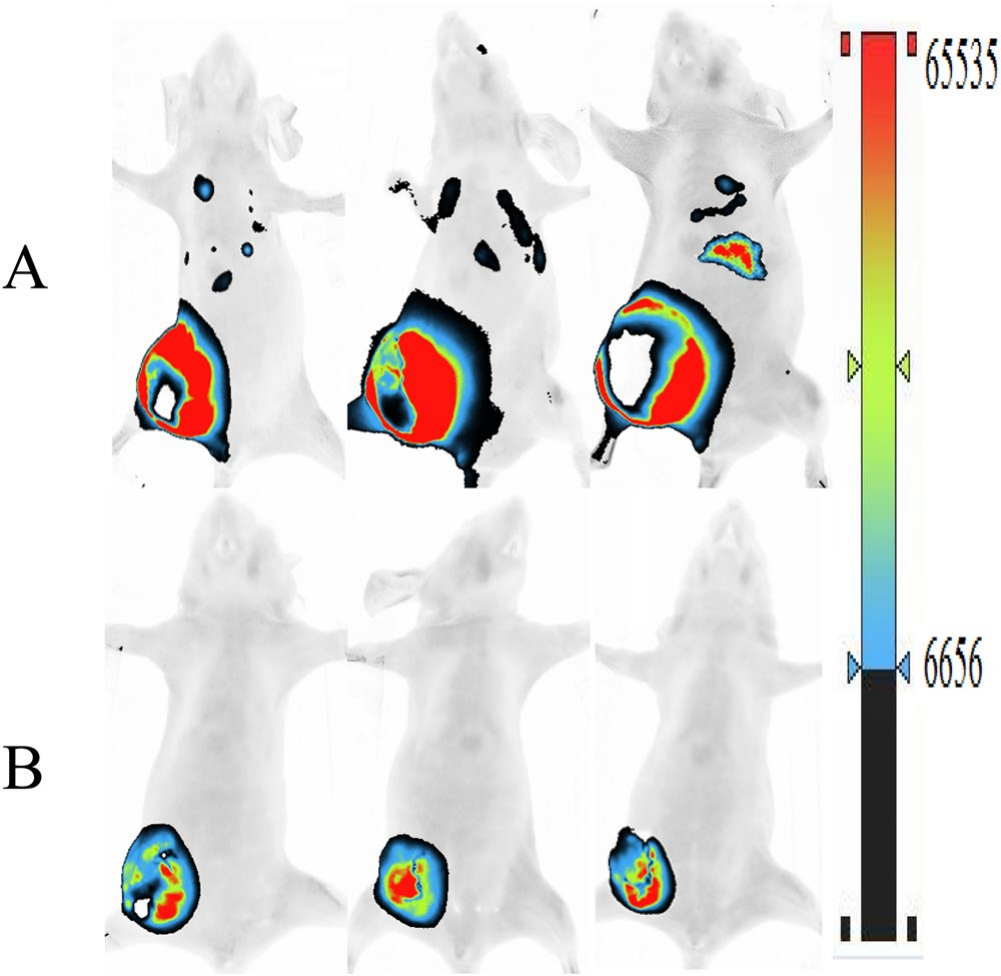
SPG-56 supressed the metastasis of 4T1 cell in nude mice. (**A**) 4T1 tumor control (4T1-TC) and (**B**) SPG-56 with high dose of 240 mg/kg body weight (4T1-SPG). 20 days after inoculation, the distribution of luminescence in the mice were detected by VILBER FUSION FX7 Spectra multifunctional imager at a wavelength of 560 nm. The closer to the color of the above of the color scale means the more the number of detected 4T1-Luc tumor cells.

Subsequently, 4T1-Luc breast carcinoma cells were inoculated into nude mice, to examine whether SPG-56 inhibited the expansion and metastasis of breast cancer *in vivo*. The higher the luminescence intensity detected, the more 4T1-Luc tumor cells are present. After inoculating 4T1-Luc cells for 20 days, the tumor luminescence e in the TC group was distributed not only in the tumor at site of inoculation, but also in other tissues, including the liver and lungs (Fig. 9A). This suggests that metastasis of the tumor occurred. However, the luminescence in the high dose of SPG-56 group was greatly reduced, existing only in the inoculated tumor area. This indicates that SPG-56 treatment controlled the growth of the tumor, and effectively inhibited metastasis.

### SPG-56 Inhibited Inflammatory Factors in MCF-7 Bearing Nude Mice

**Figure 10:**
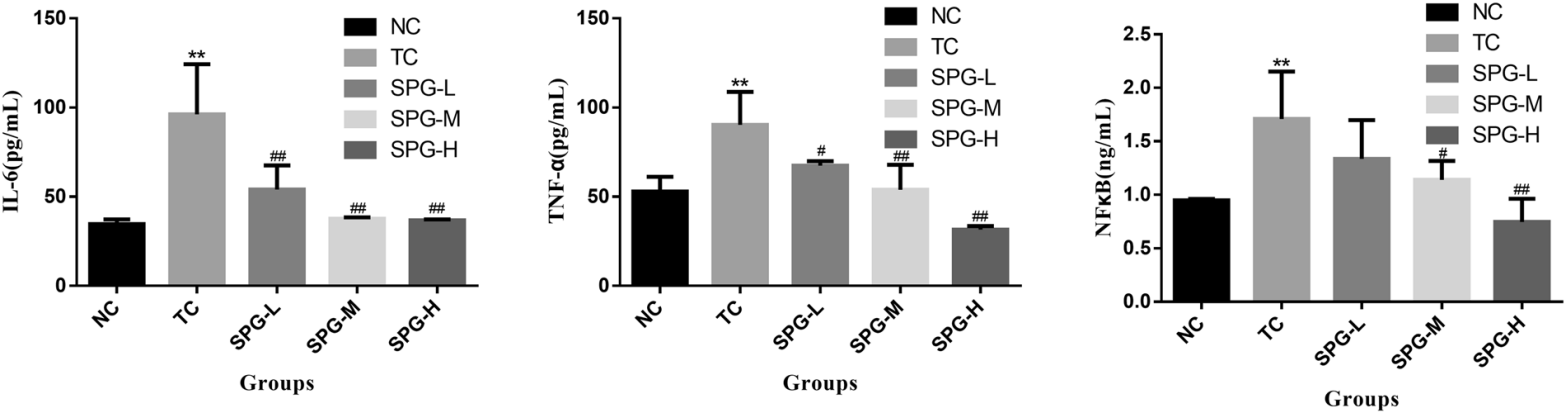
The expression of inflammatory factors in the serum. (**A**) IL-6; (**B**) TNF-α; (**C**) NFκB; *P < 0.05, **P < 0.01, compared with the NC group. ^#^P < 0.05, ^##^P < 0.01, n = 10, compared with the TC group.

In Fig. 10, compared with the NC group, the IL-6, TNF-α and NFκB levels in the serum of the TC group mice was increased by 177.7%, 70.5%, and 80.3% (P < 0.01) respectively, while the tumor marker levels were increased to a modest degree. When the lowest dosage of SPG-56 was applied to treat the tumor mice for 4 weeks, the IL-6 and TNF-α levels showed improvement, while the levels of NFκB did not differ significantly from those of the TC group. The middle and high doses of SPG-56 had significant efficacy in decreasing inflammatory factors in the serum. For the middle dose group, the IL-6, TNF-α and NFκB levels were reduced by 60.8%, 40.2% and 33.2% respectively (P < 0.05), compared with the TC group. After treatment with the highest dosage of SPG-56, the IL-6, TNF-α and NFκB was reduced by 61.7%, 65.1% and 56.3% respectively, which constituted a highly significant difference (P < 0.01) compared with the TC group. Based on these results, SPG-56 has been proved to be significantly effective in reducing the levels of inflammatory factors in the serum caused by breast cancer.

## Discussion

Breast cancer is the second most common type of cancer in the world, and the leading type in terms of morbidity and mortality in women. At present, the most effective treatments for breast cancer are surgical removal of the breast by surgery and chemotherapy. Even after these treatments, however, the risk of recurrence or metastasis is still high.

The tumor markers CEA, CA125 and CA153 are widely used in the clinical diagnosis of breast cancer, and can also be used as prognostic indicators^22,23^. In addition, these indicators have a significant correlation with the metastasis of breast cancer^24^. In our study, compared with the NC group, the serum tumor markers CEA, CA125 and CA153 were increased by 109.1%, 3855.4%and 6800.0% in the TC group. After treatment with 240 mg/kg/d of SPG-56, these three markers were decreased by 54.8%, 91.8% and 90.3% respectively, as compared with the TC group. Based on the clinical application of serum tumor markers in tumor diagnosis, it could be inferred from our experimental results that the dangers of breast cancer and its ability to metastasize were effectively reduced.

The *in vivo* experiment made use of *in situ* injection of tumor cells into the nude mouse mammary fat pad. Compared to subcutaneous inoculation, this inoculation method can better simulate the growth of breast cancer in its usual environment. The results of a preliminary experiment showed that compared with the NC group, the level of the serum tumor marker CEA in mice with subcutaneous tumors was relatively low, compared to the levels in mice inoculated *in situ*.

The *in vitro* experiment showed that SPG-56 inhibited the proliferation of MCF-7 cells in a time- and dose- dependent manner. The *in vivo* study also showed a significant suppressive effect of SPG-56 on tumor growth. At the same time, judging by to the effects of SPG-56 on HBL-100 cells, it can be concluded that the damaging effect of SPG-56 on HBL-100 cells might be negligible. The flow cytometry analysis and Hoechst 33342 staining showed that the anticancer mechanism of SPG-56 on MCF-7 cells is related to cancer apoptosis. The western blot assay and immunohistochemistry studies showed that the expression of Caspase-3, Caspase-8, and Bax in tumor tissues were significantly increased in a dose-dependent manner, while the expression of Bcl-2 was undetectable. This suggests a mitochondrial pathway for tumor apoptosis induction.

Regardless of the type of cancer, the leading cause of death is infiltration and metastasis. Breast cancer especially is prone to liver metastasis, bone metastasis, etc. Matrix metalloproteinases (MMPs) are key tumor progression promoters: a family of zinc-dependent extracellular endoproteases capable of degrading the extracellular matrix and thereby altering the basement membrane^25^. Pathologically, MMPs, which can degrade extracellular collagen, including the crucial IV, V-type collagen and gelatin in the extracellular matrix, are important in promoting tumor angiogenesis, thereby creating and maintaining a favorable environment for the growth of primary and metastatic tumors^26^, and are overexpressed in a variety of malignancies, including breast cancer. The deletion of Claudin, a membrane protein component of tight junctions, results in the formation of tumors^27^, while the overexpression of Claudin can lead to metastasis of existing tumors. Another type of tight junction protein is Occludin, which has a strong inhibitory effect on RAFL-mediated tumorigenesis. Martin *et al*. analyzed breast tumor samples and normal breast tissue samples, and found that the expression of Occludin in tumor tissue was reduced^28^. Osanai *et al*. found that Occludin can inhibit the invasiveness and movement of cancer cells, thereby reducing the metastasic potential of cancer cells^29^. Evidence suggests that an increase in STAT3 may up-regulate the expression of VEGF and other factors, thereby promoting angiogenesis and promoting tumor growth and metastasis^30^. Also evidence demonstrates that inflammation participates in providing conditions that trigger cancer^31,32^. In contrast, STAT3 signaling pathways can also inhibit MMP expression and tumor metastasis. Furthermore, IL-6 can promote tumor angiogenesis by promoting the expression of VEGF and bFGF, and can also affect the growth of tumor cells by attracting more aggressive cells to the site of the tumor. High levels of tumor necrosis factor-α can lead to systemic and immune dysfunction through multiple pathways, thereby promoting tumor cell invasion and metastasis^33,34^. Moreover, several studies have shown that nuclear factor-κB (NFκB) activity is associated with inhibition of angiogenesis, invasion and metastasis^35^. The western blot assay and immunohistochemistry studies showed that the expression of MMP9, MMP2, VEGF and STAT3 in tumor and liver tissues were significantly decreased in a dose-dependent manner. The western blot assay also showed that the expression of Occludin and Claudin in tumor and liver tissues were changed toward their normal levels. Therefore, we speculate that SPG may be able to inhibit breast cancer metastasis through several pathways.

The *in vivo* experiments support the conclusion that SPG-56 can effectively inhibit the growth and metastasis of MCF-7 breast cancer cells. These findings highlight the ability of SPG-56 to delay breast cancer development and metastasis. Therefore, SPG-56-based therapeutics have the potential to extend the lifespan of patients with breast cancer.

## Electronic supplementary material

Supplementary Material
