# Supplementary material for: Anti-breast Cancer Activity of SPG-56 from Sweet Potato in MCF-7 Bearing Mice in Situ through Promoting Apoptosis and Inhibiting Metastasis
Source: Sci Rep. 2019 Jan 16;9:146. doi: 10.1038/s41598-018-29099-x (PMC6335419; doi:10.1038/s41598-018-29099-x)
Supplement: Supplementary file 1 — Supplementary Material [file 41598_2018_29099_MOESM1_ESM.docx]

**Anti-breast Cancer Activity of SPG-56 from Sweet Potato in MCF-7 Bearing Mice *in Situ* through Promoting Apoptosis and Inhibiting Metastasis**

Zhaoxing Li^1, 2 +^, Yang Yu^1 +^, Meimei Wang^1^, Heshan Xu^3^, Bing Han^1^, Pu Jiang^1^, Hang Ma^1^, Yuanfeng Li^3^, Cheng Tian^1^, Deqi Zhou^4^, Xuegang Li^1*^, Xiaoli Ye^3*^

1 School of Pharmaceutical Sciences, Southwest University, Chongqing, 400716, China

2 McLean Hospital, Harvard Medical School, Belmont, 02478, MA, USA

3 Chongqing Engineering Research Centre for Sweet Potato, School of Life Sciences, Southwest University, Chongqing, 400715, China

4 Oncology Department, Chongqing Beibei District Hospital of Traditional Chinese Medicine, Chongqing, 400700, China

+ These authors contributed equally to this study.

* Corresponding author

School of Pharmaceutical Sciences, Southwest University, Chongqing, 400715, China. Tel: +86 023-68250728; Fax: +86 023-68250728. E-mail addresses: [Xuegangli@swu.edu.cn](mailto:Xuegangli@swu.edu.cn).

Chongqing Engineering Research Centre for Sweet Potato, School of Life Sciences, Southwest University, Chongqing, 400715, China. Tel: +86 023-68250728; Fax: +86 023-68250728. E-mail addresses: [yexiaoli@swu.edu.cn](mailto:yexiaoli@swu.edu.cn).

E-mail addresses: zli@mclean.harvard.edu (ZX. Li), [798627878@qq.com](mailto:798627878@qq.com) (Y. Yu), wmm543@126.com (MM. Wang), 347504110@qq.com (HS Xu), hanbing7525@163.com (B. Han), 943634044@qq.com (P. Jiang), 20116806@qu.edu.cn (H. Ma), 1010661096@qq.com (YF. Li), 2269315199@qq.com (C. Tian), [18908355177@163.com (DQ](mailto:18908355177@163.com%20(DQ). Zhou)

**Supplementary details about SPG-56**

**1. Material source**

The SPG-56 was extracted from fresh hybrid sweet potato variety Zhongshu-1, which was obtained from the Sweet Potato Research Center of Chongqing, China. And its profile is showed in Fig. A.

**2.** **Separation method**

SPG-56 separation process shown in Fig. B. Wang named fr.17 as SPG-56.

**3. Amino acid and sugar compositions of SPG-56**

The monosaccharide analysis of SPG-56 was shown in Fig. C.

Total sugar contents of SPG-56 were measured by sulfuric acid-anthrone colorimetry. The results showed that the sugar content was about 2.9%. Further analysis of SPG-56 by HPLC displayed that the types of monosaccharides was up to six, consist of mannose, glucuronic acid, galacturonic acid, xylose, galactose, and arabinose. The contents of these monosaccharides were about 0.21%, 0.17%, 0.20%, 0.23%, 0.82% and 0.92%, respectively.

The total content of protein in SPG-56 was about 97.1% determined by Coomassie brilliant blue method. The results of biological mass spectrometry indicated that the molecular weight of SPG-56 was 56 KD, which was shown in Fig. D. The contents and the composition of amino acids in SPG-56 were determined by the analysis of automatic amino acid analyzer, as shown in Table 2. It was discovered that the SPG-56 contained 15 kinds of amino acids, of which 7 kinds of essential amino acid was up to 42.9%. Interestingly, glutamic acid, aspartic acid and valine were abundant in SPG-56, and their contents were about 14.2%, 14.0% and 9.5%, respectively.

All figures in the supplement were provided by Wang Meimei.

The authors declare no competing interests.


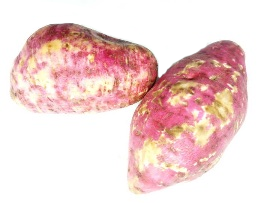

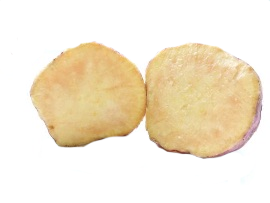


Fig. A Profile of new hybrid sweet potato variety Zhongshu-1 from the Sweet Potato Research Center of Chongqing, China.

Fig. B SPG-56 separation process


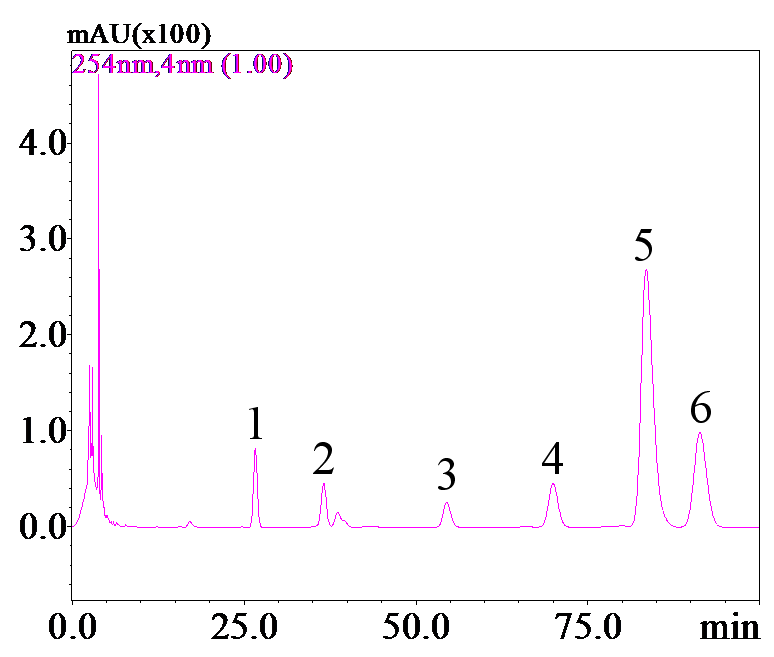


Fig. C Monosaccharides analysis of SPG-56, performed by high performance liquid chromatography(HPLC). 1, mannose; 2, glucuronic acid; 3, galacturonic acid; 4, xylose; 5, galactose; 6, arabinose.


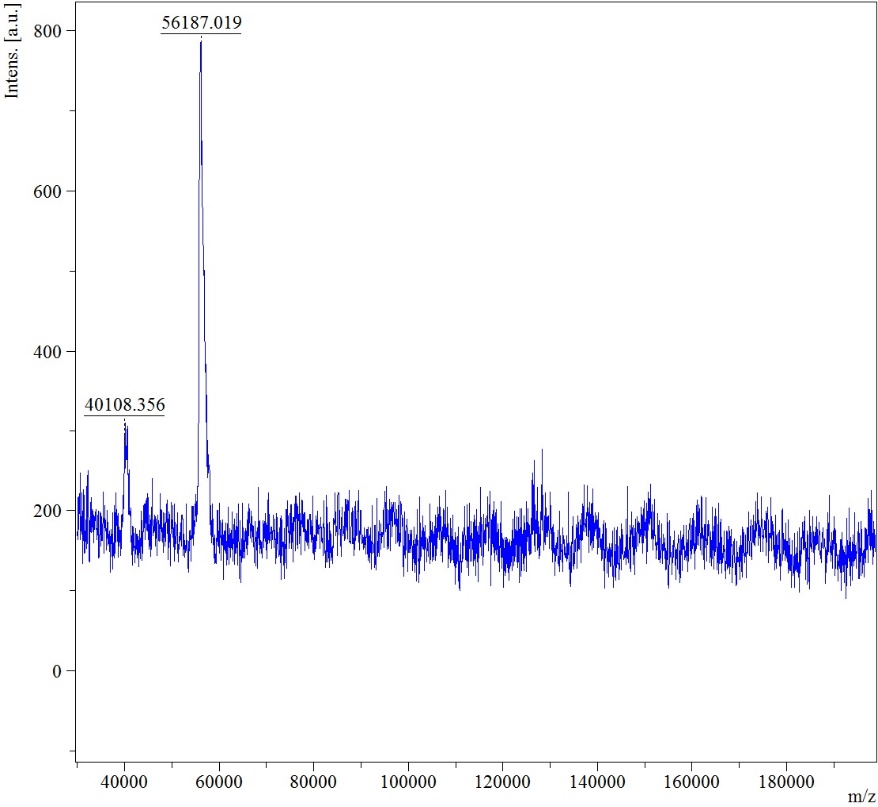


Fig. D. Molecular weight detection of SPG-56 by a biological mass spectrometry.
